# Epigenetic Regulation of Glycosylation in Cancer and Other Diseases

**DOI:** 10.3390/ijms22062980

**Published:** 2021-03-15

**Authors:** Rossella Indellicato, Marco Trinchera

**Affiliations:** 1 Department of Health Sciences, University of Milan, 20142 Milan, Italy; 2 Department of Medicine and Surgery, University of Insubria, 21100 Varese, Italy; marco.trinchera@uninsubria.it

**Keywords:** epigenetics, methylation, histone acetylation, miRNAs, glycosylation, glycogenes, cancer, inflammatory bowel disease, IgA nephropathy

## Abstract

In the last few decades, the newly emerging field of epigenetic regulation of glycosylation acquired more importance because it is unraveling physiological and pathological mechanisms related to glycan functions. Glycosylation is a complex process in which proteins and lipids are modified by the attachment of monosaccharides. The main actors in this kind of modification are the glycoenzymes, which are translated from glycosylation-related genes (or glycogenes). The expression of glycogenes is regulated by transcription factors and epigenetic mechanisms (mainly DNA methylation, histone acetylation and noncoding RNAs). This review focuses only on these last ones, in relation to cancer and other diseases, such as inflammatory bowel disease and IgA1 nephropathy. In fact, it is clear that a deeper knowledge in the fine-tuning of glycogenes is essential for acquiring new insights in the glycan field, especially if this could be useful for finding novel and personalized therapeutics.

## 1. Introduction

In the vast universe of cell biology, there is a very elaborate mechanism capable of carrying out a myriad of functions: glycosylation of proteins and lipids. It consists of the enzymatic attachment of monosaccharides to lipid or protein molecules [1], giving rise to a class of macromolecules called the glycoconjugates (glycoproteins, proteoglycans, mucins, glycosphingolipids, lipopolysaccharides). Glycoconjugates differ in their glycan (the carbohydrate chain) sequence, length, number and position of branches, and type of connections between sugars [1,2]. The complexity of glycan structures is due to the fact that glycan synthesis is not template-driven, unlike linear molecules such as DNA and proteins [3], and is influenced by many variables, including environmental factors, genetic factors (i.e., single nucleotide polymorphisms), transcription factors, protein transports, altered pH values in subcellular sites (especially in the Golgi apparatus), Golgi organizers, ion channels, oxygen concentration, subcellular localization of enzymes, activated monosaccharide donor substrates, and acceptor substrates availability [3,4,5]. In fact, it is estimated that more than 800 genes are involved in the process of glycosylation [6,7], and, among these, about 500 glycosylation-related genes (or glycogenes) are directly involved in glycan assembly, remodeling and degradation, and account for about 2% of the genome [8,9].

Glycosylation has important implications for numerous processes, including the following.

Physical and structural role: a dense layer of glycans (glycocalyx) covers the surface of all the cells allowing to modulate cell–cell, cell–matrix, and cell–molecule interactions, critical to the development of a complex multicellular organism [10,11]; protein folding [9]: it takes place in the endoplasmic reticulum and ensures protein stability and functioning [10]; transcriptional regulation [9,12]: *O*-GlcNAcylation participates in the epigenetic regulation of gene expression [12,13,14]; interactions between host and pathogenic microorganism [10,11]: viruses and bacteria bind and get access to host cells through glycan receptors [15,16] and are able to evade the immune system decorating themselves with a layer of host-like glycans (the so-called “molecular mimicry” mechanism) [9,17]. Parasites use another strategy to survive, called “glycan gimmickry”, consisting in targeting host–glycan-binding proteins with their glycans [18].

One of the most important features of glycosylation is that it takes place in a cell- and tissue-specific manner [3,6,19]. For example, it plays a very critical role in the development and function of the nervous system, where characteristic glycan structures regulate axon pathfinding, neurite outgrowth, synaptogenesis, neurotransmission, and other neuronal processes [20]. Moreover, neural-specific glycans are required to carry out high-order brain functions, including learning/memory and the formation of neural networks [6,21].

This review briefly describes the principal epigenetic mechanisms that participate in gene expression and how they are related to glycosylation. A short overview illustrates the normal physiology of epigenetic regulation of glycogenes, and a more extensive section is devoted to cancer and other diseases.

## 2. Epigenetic Regulation of Glycosylation

Since glycosylation is cell- and tissue-specific, but the DNA template is always the same in every cell of an organism, a master player able to regulate gene expression is required: epigenetics. It was defined as a “stably heritable phenotype resulting from changes in a chromosome without alterations in the DNA sequence” [22]. It is a process that takes place during the differentiation of somatic cells, as well as in response to environmental changes [23]. Epigenetics is one of the reasons why the cells of an organism have a different phenotype, even if they share the same DNA sequence. Epigenetics acts in order to regulate gene expression mainly through the following three mechanisms [24,25,26,27,28].

DNA methylation: it occurs in CpG rich-regions called CpG islands, where CpG dinucleotides tend to cluster. Frequently, methylation of these regions represses gene transcription and expression [29], while unmethylated regions promote gene activation. Basically, methylation requires a methyl group (CH_3_) covalently attached to the 5-carbon of the cytosine residue (5mC) in the CpG site [26,30,31]. This action is carried out by DNA methyltransferases (DNMTs), and the CH_3_ group physically interrupts the binding between the proper transcription factor and its recognition sequence. Moreover, gene silencing upon methylation can also occur when methyl-CpG-binding proteins bind to the methylated DNA and recruit co-repressor molecules, such as histone deacetylases, to induce chromatin structure condensation [32].

Histone modifications: the two main modifications that can occur on histones are methylation and acetylation. They alter chromatin structure; in fact, euchromatin (actively transcribed) is characterized by high levels of acetylation and di/trimethylation of H3K4, H3K36 and H3K79 [29,33], while heterochromatin (transcriptionally inactive) is characterized by low levels of acetylation and high levels of H3K9, H3K27 and H4K20 methylation [29]. *O*-GlcNAcylation is another form of histone modification [12,13,14], and it is the perfect example of how epigenetics and glycosylation are tangled together: epigenetics regulates glycogenes expression, and glycosylation participates in epigenetic regulation.

Noncoding RNAs (ncRNAs): a wide range of RNA molecules belongs to this category, which is included transfer RNA (tRNA), ribosomal RNA (rRNA), microRNA (miRNA), small interfering RNA (siRNA), piwi-interacting RNA (piRNA), small nuclear RNA (snRNA), small nucleolar RNA (snoRNA), and long noncoding RNA (lcnRNA) [34,35,36]. The most studied are miRNAs and lncRNAs. miRNAs are characterized by a short sequence of nucleotides (about 20–30), play a role in gene silencing [31] targeting mRNA 3’UTR regions, and inhibit protein translation or enhance mRNA degradation [26,34]. To date, over 80 glycogenes have been identified as miRNAs targets [37]. LncRNAs are longer than miRNAs (up to more than 100 kilobases) [25] and can function both by repressing or activating gene expression [34], acting as molecular chaperones or scaffolds for various chromatin regulators [31].

Of course, epigenetics is not the only mechanism involved in the transcription of glycogenes. In fact, also transcription factors binding to a gene promoter and enhancer elements are fundamental for this purpose [38]. A prominent example is given by transcription factors hepatocyte nuclear factor 1α (HNF1α) and its downstream target hepatocyte nuclear factor 4α (HNF4α), which were proven by Lauc et al. in the first genome-wide association study (GWAS) of protein glycosylation to regulate the expression of key fucosyltransferases and fucose biosynthesis genes. This finding revealed a new role for HNF1α as a master transcriptional regulator of multiple stages in the fucosylation process [39].

## 3. Physiological Aspects of Epigenetic Regulation of Glycosylation

Before going deeper into the field of epigenetic regulation associated with pathological glycosylations, it is worth making a brief presentation on how glycogenes are regulated by epigenetics when it comes to normal physiology. Research in this field is just at the beginning, and there are little available data at present. Yet, some prominent studies carried out on the brain elucidated the significance/importance of specific neural glycans since their fine-tuning is pivotal for high-order brain functions (i.e., learning/memory, the formation of the neural network, myelination), and their dysregulation leads to various neurological disorders [40]. The first research group focused on a glycosyltransferase called N-acetylglucosaminyltransferase IX (MGAT5B), that catalyzes the transfer of *N*-acetylglucosamine (GlcNAc) to the 6-OH position of the mannose residues of GlcNAcβ1,2-Manα on both the α1,3- and α1,6-linked mannose arms in the core structure of *N*-glycans. It is also responsible for the transfer of GlcNAc in β1,6-linkage to *O*-mannosyl glycans. The gene encoding this enzyme is *MGAT5B*, which is exclusively expressed in the brain [41], and it has been proved that it is under the control of neural cell-specific histone modification: active chromatin marks like H3K9ac and H3K4me3 were found in the mouse brain, and repressive chromatin marks like H3K27me3 and H3K9me2 were detected in mouse kidney and liver [42]. The second research group studied two glycosyltransferases involved in lipid glycosylation: B4GALNT1 and ST8SIA1. They are both involved in the biosynthesis of gangliosides, a class of sialic acid-containing glycosphingolipids particularly abundant in the central nervous system. Their peculiarity consists in being ontogenically regulated, and in fact, they are more expressed in the adult brain. Experiments on mice showed that brain gangliosides shift from the simpler ones (GM3 and GD3) in early phases of life to more complex ones during development (GM1, GD1a, GT1a, and GT1b) and that expression of B4galnt1 (prevalently) and St8Sia1, both involved in this shifting, increased, due to histone H3 and H4 acetylation [43,44,45].

## 4. Epigenetic Regulation of Glycosylation in Cancer

The majority of the studies of epigenetic regulation of glycogenes are about cancer. It is well-established that aberrant glycosylation is one of the hallmarks of tumoral cells [46,47,48] and that these changes are nonrandom: in cancer advancement, only the fittest cells survive, and specific glycan changes are selected for tumor progression [47]. In fact, transcription of a gene tends to be constitutively repressed in cancer, when its epigenetic silencing is advantageous for promoting cancer progression [49]. In particular, incomplete synthesis and neo-synthesis processes are the two principal mechanisms associated with alterations of carbohydrate structures during tumor progression [50]. Incomplete synthesis refers to truncated glycosylation that produces the Tn antigen in mucin-type *O*-glycans, and neo-synthesis produces abnormal glycosylation patterns such as sialyl Lewis X (sLex) [51,52]. Tn antigen and sLex are typical of lymphocytes and help in their extravasation from the blood, while in cancer, they facilitate metastatic spread [1,53]. Novel glycan structures also have the role of enabling cancer cells to evade the host immune response [15,51,54,55].

All these modifications in glycosylation during the tumoral event are carried out by genetic, epigenetic, metabolic, inflammatory and environmental mechanisms [52], but this review focuses only on epigenetic alterations that affect glycogenes during carcinogenesis. The first studies were based on the methylation status of the promoter region, using demethylating agents such as 5-aza-2-deoxycytidine (5-aza-dC) [56,57,58]. Later on, it was discovered that hypermethylation of a promoter could not be sufficient to maintain gene silencing; in fact, even upon a treatment, only a partial restoration was achieved, and this was due to other epigenetic marks involved such as repressive histone modifications [59,60].

Epigenetic modifications of glycogenes in cancer were extensively reviewed by Dall’Olio and Trinchera [48], and the most recent ones are updated in Table 1, but it is most likely that the number of glycogenes epigenetically regulated in cancer is going to grow in the next years.

Below, we present some prominent examples of epigenetically modified glycogenes involved in tumor progression.

### 4.1. C1GALT1C1

One of the hallmarks of carcinoma mucins is their incomplete glycosylation. The addition of the first *N*-acetylgalactosamine (GalNAc) *O*-linked to serine or threonine of mucin-type glycans leads to the formation of the Tn antigen, which is a well-known cancer-associated structure [48]. On this first GalNAc, a Gal residue could be added by core 1 β1,3-galactosyltransferase (C1GALT1 or T-synthase), which needs the molecular chaperone C1GALT1C1 (encoded by *C1GALT1C1* gene) for its functioning. This leads to the formation of the T antigen. At the same time, another enzyme called sialyltransferase ST6GALNAC1 could act on the Tn antigen, adding a residue of α2,6-linked sialic acid, resulting in the formation of the sialyl-Tn (STn) antigen and blocking further chain elongation [52,104]. During carcinogenesis, C1GALT1C1 expression could be downregulated due to genetic mutations [105] or, more interestingly, to an epigenetic modification: hypermethylation of the promoter leads to the silencing of C1GALT1C1 and to the accumulation of the cancer-associated Tn and STn antigens [106,107]. The secreted mucins expressing these antigens often appear in the bloodstream of patients with cancer and are associated with invasion since they potentiate migration of tumor cells through the inhibition of cell–cell contacts [108,109]. Moreover, these carcinoma mucins often decorate the tumor surface, creating clustered sites for antibody attachment, thereby improving their activity as tumor immunogens. In fact, since these glycans infrequently occur in normal tissues, they provoke immune responses in patients, a property that has been exploited for potential immunotherapy [47,108].

### 4.2. B4GALNT2 (β-1,4-N-acetyl-galactosaminyltransferase 2)

Sda carbohydrate (GalNAcβ1,4[Sialα2,3)Galβ1,4GlcNAc) belongs to the category of the histo-blood group antigens. They were initially found on the erythrocyte surface, but it was soon discovered that this group of antigens is widely distributed in many epithelial tissues (colon, stomach, kidney, oocyte) and secretions (urine, serum, saliva, milk) [110,111,112], and play roles in the regulation of physiological mechanisms. In particular, studies in murine models showed that the Sda antigen is involved in the processes of hemostasis [113,114] and reproduction [115,116]. The last step in the biosynthesis of the Sda antigen is catalyzed by GalNAc transferase B4GALNT2 (also known as Sda synthase), which adds an *N*-acetylgalactosamine to a terminal α2,3-sialylated galactose residue [117]. Experiments on guinea-pigs [118] and rats [119] proved that the *B4GALNT2* gene could be ontogenically regulated; in fact, the enzyme was absent at birth and increased with age. More information on Sda synthase is known as far as concern cancer since the expression and activity of this enzyme are downregulated in gastrointestinal cancer leading to a complete loss of the antigen [112,117,120]. The reason for such differential expression was attributed to the hypermethylation of the *B4GALNT2* promoter [120,121], which is embedded in CpG islands. In work by Kawamura et al. [54], the *B4GALNT2* gene was found methylated in about one-half of the gastric cancer cases taken under consideration and in the majority of gastric and colon cancer cell lines. They used a demethylating agent, 5-aza-dC, to attempt a recovery of *B4GALNT2* transcription, but it worked only partially, inducing a very weak expression of both the glycoenzyme and the Sda antigen. Human colon cancer cells were also treated with the histone acetylase inhibitor butyrate, but neither a slight recovery of the Sda antigen nor that of B4GALNT2 was observed. According to these results, the mechanism of *B4GALNT2* downregulation in cancer deserves further investigation [122].

### 4.3. B3GALT5

B3GALT5 is one of the glycoenzymes involved in the synthesis of type 1 chain carbohydrate antigens, namely the Lewis a (Lea) trisaccharide, the Lewis b (Leb) tetrasaccharide and the sialyl Lewis a (sLea) tetrasaccharide [123,124]. Lea and Leb are involved in various biological contexts, such as microbial adhesion and cancer [125], whereas sLea has been proven to be specifically an E-selectin ligand, favoring the metastatic process and angiogenesis during cancer development [124,126,127]. The peculiarity of B3GALT5 is that its expression is regulated by two promoters: the LTR and native promoters [128].

The LTR promoter, which has retroviral origins and is activated through hepatocyte nuclear factor HNF1α and HNF1β [129,130], is mainly active in the organs of the gastrointestinal tract (such as the colon, stomach, and pancreas). However, HNF1α and HNF1β are not able to modulate transcription, which depends on distal regulatory elements that are active when methylated. In fact, LTR and proximal sequences lack CpG islands, suggesting that methylation-sensitive DNA sequences reside outside the LTR region, presumably distant from the promoter, where they act as potential epigenetic regulators of transcription [130,131].

In the mammary glands, thymus and trachea, as well as in some human cancer cell lines, transcription is mainly driven by a native promoter, which is sensitive to nuclear factor NF-Y [124] and is located nearby two CpG islands [132] epigenetically regulated through methylation [130]. As for the LTR promoter, NF-Y is unable to regulate transcription, which depends on the methylation of the regulatory elements [130,131]. Moreover, histone modification is another mechanism involved in the regulation. High expression of the native transcript is associated with active histone marks (H3K4me3, H3K79me2, H3K9Ac, and H3K9-14Ac), while low levels of the transcript are associated with repressive histone marks (H3K27me2 and H4K20me3) [132].

The differential regulation of *B3GALT5* was studied in particular in the pancreas and colon, comparing normal and tumoral tissues [130,131,132]. B3GALT5 is strongly downregulated in colon cancer with respect to the normal mucosa [133,134], and the silencing of the gene is due to the opposite but synergic behavior of the two promoters: hypomethylation of the distant sequences of the LTR promoter and hypermethylation of the native promoter [124]. In the pancreas, both normal and cancer tissues have very low levels of methylation in the native promoter, and the levels of B3GALT5 LTR transcript were similar to those of the native transcript, without difference between normal and tumoral specimens [131,132].

## 5. Epigenetic Regulation of Glycosylation in Other Diseases

Dysregulation of glycosylation is associated not only with cancer but also with a number of other diseases. The majority of them are caused by genetic mutations such as congenital disorders of glycosylation, diabetes, cardiovascular, immunological, autoimmune (rheumatoid arthritis, Sjögren’s syndrome, systemic lupus erythematosus) and infectious disorders [2,7]. Other diseases are associated with both genetic and epigenetic modifications, such as inflammatory bowel disease (IBD), IgA1 nephropathy (IgAN), and neurodegenerative diseases, briefly reviewed below. Since this is a recent field of research, it is highly probable that the disorders associated with aberrant epigenetic regulation of glycosylation will increase over time, giving a better insight into the disease pathogenesis.

### 5.1. Inflammatory Bowel Disease

IBD is a chronic inflammatory disorder that affects the gastrointestinal tract and comprises two clinical syndromes: Crohn’s disease (CD) and ulcerative colitis (UC) [135,136]. These diseases have unknown etiology, and there is insufficient information about pathogenesis, but it is believed that a complex interaction of genetic, epigenetic, microbial, environmental and immunological factors are involved [137]. In particular, several studies have evaluated the epigenetic status of IBD patients using candidate gene strategies [138,139,140,141,142] or epigenome-wide association studies [143,144,145,146], trying to elucidate IBD pathogenesis [147]. Cooke and colleagues [143] collected rectal biopsies and identified some glycogenes that have a differential methylation status between patients with CD and UC (inflamed vs. non-inflamed) and healthy controls. In fact, in inflamed UC vs. controls, B3GALT2, GFPT1 and GBGT1 have increased methylation; in inflamed CD vs. controls, GFPT1 and GBGT1 have increased methylation and FUT2 has a decreased methylation; in non-inflamed CD vs. controls, FUT7 and FGF23 have decreased methylation. These altered methylation levels correlated with the development of IBD, contributing to better understand IBD pathogenesis. Another study conducted by Klasíc and colleagues evaluated the methylation status of β-1,4-mannosyl-glycoprotein 4-β-*N*-acetylglucosaminyltransferase (*MGAT3*) promoter in CD3+ T cells isolated from the inflamed mucosa of UC patients. They found that the *MGAT3* promoter was hypermethylated in UC patients compared with healthy controls. This kind of deregulation might lead to an increase of the proinflammatory properties of IgG through a decrease in galactosylation and sialylation and an increase of bisecting GlcNAc on digalactosylated glycans, thus suggesting a functional role of *MGAT3* in IBD pathogenesis [148].

### 5.2. IgA1 Nephropathy

Several studies led to the conclusion that inhibition of genes involved in glycosylation by miRNAs plays a role in the pathogenesis of IgA1 nephropathy (IgAN), which is characterized by the aggregation of aberrantly glycosylated IgA1 molecules, leading to the synthesis of inflammatory cytokines and glomerulonephritis. The first study conducted by Serino and colleagues brought to the attention the role of miR-148b. It was demonstrated that peripheral blood mononuclear cells (PBMCs) of patients with IgAN show a higher miR-148b expression level compared to healthy controls, and this upregulation leads to a lower C1GALT1 expression. C1GALT1 is involved in the *O*-glycosylation of the IgA1 heavy chain hinge-region, and without the expression of the gene, hinge-region displays a deficiency of galactose [149]. This group also demonstrated that GALNT2 (UDP-GalNAc: polypeptide *N*-acetylgalactosaminyltransferase 2) is the target of miRNA let-7b, similarly to C1GALNT1 and miR-148b. GALNT2 initiates the addition of GalNAc to serine or threonine residues of the IgA1 hinge-region. Let-7b was significantly upregulated in IgAN patients, and, as a consequence, GALNT2 levels became lower [150].

Recently, another miRNA was found to be involved in the aberrant glycosylation of IgAN, but not in a direct way. In fact, the direct target of miR-98–5p is CCL3 (C–C motif chemokine ligand 3), which can change the level of Th1 and Th2 cytokines in many diseases. Th1 and Th2 cytokines participate in the pathogenesis of IgAN. In this case, only IL-6 was found to be upregulated in PBMCs of IgAN patients compared to healthy controls. IL-6 reduces the galactosylation of IgA1 by decreasing the expression of C1GALT1 [151].

Another miRNA able to indirectly modify glycosylation in IgAN is miR-374b. The target of this miRNA is C1GALT1C1, which is required for the activity of C1GALT1, and that is downregulated in the B cells isolated from IgAN patients, leading to abnormal glycosylation of IgA1 [152]. Furthermore, miR-320 (upregulated in the renal tissues of IgAN patients) targets C1GALT1C1, which in fact is downregulated in the same patients [67].

### 5.3. Neurodegenerative Diseases

A critical role of glycosylation is emerging in the field of neuron homeostasis and related neurodegenerative diseases. It is well-known that several glycoconjugates and related processing enzymes, namely glycosyltransferases and glycosidases, are strictly and specifically expressed in the central nervous system, and a set of specific glycosylations, such as ganglioside biosynthesis and GlcNAcylation, are strongly associated with various neurodegenerative disorders [153]. At present, the majority of data arise from genetic defects. Both KO mice of ganglioside glycosyltransferases and congenital disorders of glycosylation affecting ganglioside biosynthesis indicated that ganglioside dysregulation gives rise to neuroinflammation, functional impairment, and in turn neurodegeneration [153,154]. Moreover, non-genetic derangement of glycosyltransferases was associated with Parkinson’s disease (reduced B3GALT4 and ST3GAL2, increased OGT, O-linked GlcNAc transferase), Huntington disease (reduced ST3GAL5, ST3GAL2, ST8SIA3, B4GALNT1), Alzheimer’s disease, and even amyotrophic lateral sclerosis (general ganglioside overexpression) [153,155]. In multiple sclerosis, an autoimmune disease causing inflammation of the central nervous system, glycoproteins are candidate targets of autoreactivity, and glycosyltransferases such as MGAT1, MGAT5 and B4GALT6 are reported as deregulated genes [7,156]. A key role of epigenetic regulation is reported in multiple sclerosis [7] and suggests that such mechanism could be the common trait of some of the other neurodegenerative disorders associated with deranged glycosylation.

## 6. Concluding Remarks

Epigenetic regulation of glycosylation is an emerging and relatively recent field of research. The analysis of glycogenes expression due to epigenetic mechanisms started with the use of demethylating agents in cancer cell cultures [57,157], and it has become more important over the years. At present, several pathological mechanisms associated with cancer and other diseases are known to be caused by epigenetic dysregulation of glycosylation, as reported in this review. We also reported relevant studies illustrating how epigenetics controls glycosylation under physiological conditions [3,6,44,45,122,158,159]. Altogether these findings help to unravel the roles and functions of glycans, which are candidate targets in the field of personalized medicine through drugs-based inhibitors of their synthesis, glycan antagonists, and glycan-function modulators [52]. In this regard, it is also worth recalling the critical interplays involving glycosylation, epigenetics, and hypoxia since one controls the other. This suggests that drugs affecting glycosylation through epigenetic regulation could be relevant in cancer developing chemo-resistance [26].

## Figures and Tables

**Table 1:** List of glycogenes regulated through epigenetics.

Target	Epigenetic Mechanism	Effect	Tissue/Cells Involved	References
***Galactosyltransferases***				
B4GALT3	miR-1247-3p CircUBXN7/miR-1247-3p axis	Downregulation Upregulation	CAFs in lung metastasis of liver cancer Bladder cancer	[61,62]
***N-acetyl-galactosaminyl transferases***				
GALNT1	LncRNA SNHG7/miR-216b axis	Upregulation	Colorectal cancer	[63]
GALNT3	Linc01296/miR-26a axis	Upregulation	Colorectal cancer	[64]
GALNT4	miR-4262 (downregulated)	Upregulation	Colorectal cancer	[65]
GALNT7	miR-30e (downregulated) LncRNA SNHG7/miR-34a axis miR-154 (downregulated) miR-125a-5p (downregulated)	Upregulation Upregulation Upregulation Upregulation	Cervical cancer Colorectal cancer Laryngeal squamous cell carcinoma Cervical cancer	[66,67,68,69]
GALNT14	Hypermethylation	Downregulation	A549-T cells (paclitaxel-resistant strain of human non-small cell lung cancer)	[70]
B4GALNT1	Histone acetylation Hypermethylation	Upregulation Downregulation	Renal cell carcinoma Hepatocellular carcinoma	[71,72]
***N-acetyl-glucosaminyl transferases***				
MGAT3	miR-23a(upregulated)	Downregulation	Hca-P (mouse) cell line	[73]
OGT	miR-24-1 (downregulated) miR-24 (downregulated) miR-483 (downregulated) miR-485-5p (downregulated)	Upregulation Upregulation Upregulation Upregulation	Hca-F (mouse) cell line High invasive breast cancer cell lines Gastric cancer Colorectal cancer and esophageal cancer cell lines	[74,75,76,77,78]
***Sialyltransferases***				
ST3GAL4	miR-370 (treatment)	Downregulation	Colo 320 cell line	[79]
ST6GAL1	miR-9 (downregulated) LncRNA ZFAS1/miR-150 axis LncRNA HOTAIR/miR-214 axis	Upregulation Upregulation Upregulation	Hepatocellular carcinoma cell lines with high lymphatic metastatic potential T-cell acute lymphoblastic leukemia Colorectal cancer	[80,81,82]
ST6GAL2	LncRNA HCP5/miR-22-3p, miR-186-5p, miR-216a-5p axis	Upregulation	Follicular thyroid carcinoma	[83]
ST6GALNAC2	miR-182 and miR-135b	Downregulation	Colorectal cancer	[84]
ST6GALNAC3	Promoter hypermethylation	Downregulation	Prostate cancer	[85]
ST6GALNAC5	Promoter hypermethylation	Downregulation	Cervical cancer	[86]
ST6GALNAC6	Histone methylation (H3K27me3)	Downregulation	Colon cancer	[87]
ST8SIA1	miR-33a and let-7e (downregulated) Promoter hypomethylation Promoter hypermethylation	Not evaluated Upregulation Not evaluated	Colorectal cancer Triple-negative breast cancer Esophageal cancer	[88,89,90]
ST8SIA4	miR-146a and miR-146b (upregulated)miR-146a (upregulated)	Downregulation Downregulation	Follicular thyroid carcinomaOral squamous carcinoma cell lines	[91,92]
***Fucosyltransferases***				
FUT1	miR-34a (downregulated)	Upregulation	Head and neck squamous cell carcinoma	[93]
FUT4	miR-26a e miR-26b (downregulated) miR-200b (downregulated) miR-125a-5p (downregulated) miR-200c (treatment) miR-1295b and miR-6715amiR-29b/Sp1 axis LncRNA AC114812.8/miR-371b-5p axis	Upregulation Upregulation Upregulation Downregulation Not evaluated Upregulation Upregulation	Colorectal cancer Breast cancer Bladder cancer cell lines MCF-7 cell line (breast cancer) Cholangiocarcinoma Acute myeloid leukemia Bladder cancer cell lines	[94,95,96,97,98,99,100]
FUT5	miR-125a-3p (downregulated)	Upregulation	Colorectal cancer	[101]
FUT6	miR-125a-3p (downregulated) LncRNA HOTAIR/miR-326 axis	Upregulation Upregulation	Colorectal cancer Colorectal cancer	[101,102]
***Sulfotransferases***				
HS3ST3B1	miR-218 (downregulated)	Upregulation	Non-small cell lung cancer	[103]
***Nucleotide donor transporters***				
DTDST	Histone methylation (H3K27me3)	Downregulation	Colon cancer	[87]

## Data Availability

Not applicable.

## Abbreviations

Genes and proteins are named according to the HUGO recommendations.

5-aza-dC	5-Aza-2-deoxycytidine
B3GALT5	β1,3-Galactosyltransferase isoenzyme 5
B4GALNT2	β-1,4-*N*-Acetylgalactosaminyltransferase 2
C1GALT1	Core 1 β1,3-galactosyltransferase
C1GALT1C1	C1GALT1-specific chaperone 1
CAF	Cancer-associated fibroblast
CD	Crohn’s disease
FGF23	Fibroblast growth factor 23
GalNAc	*N*-Acetylgalactosamine
GBGT1	Globoside α-1,3-N-Acetylgalactosaminyltransferase 1
GALNT2	UDP-GalNAc: polypeptide N-acetylgalactosaminyltransferase 2
GFPT1	Glutamine-fructose-6-phosphate transaminase 1
GlcNAc	*N*-Acetylglucosamine
HNF1α	Hepatocyte nuclear factor 1α
HNF4α	Hepatocyte nuclear factor 4α
IBD	Inflammatory bowel disease
IgAN	IgA1 nephropathy
MGAT3	β-1,4-Mannosyl-glycoprotein 4-β-N-acetylglucosaminyltransferase
OGT	*O*-Linked GlcNAc transferase
PBMC	Peripheral blood mononuclear cell
lncRNA	Long noncoding RNA
miRNA	MicroRNA
ncRNA	Noncoding RNA
piRNA	Piwi-interacting RNA
rRNA	Ribosomal RNA
siRNA	Small interfering RNA
snRNA	Small nuclear RNA
snoRNA	Small nucleolar RNA
tRNA	Transfer RNA
sLex	Sialyl Lewis X
UC	Ulcerative colitis
